# Homœopathy Amongst the American Indians

**Published:** 1884-02

**Authors:** 


### HOMŒOPATHY AMONGST THE AMERICAN INDIANS.

Mr. Herbert Welsh, of Philadelphia, from whose report of a visit (made June, 1883) to the Santee Indians we shall presently quote, is a son of the Hon. John Welsh, ex-Minister to Great Britain, and a gentleman of undoubted veracity. The gentleman employs the "regular" profession, is not a believer in Homœopathy, hence his testimony is the more striking and unbiased! In his report he writes:

"On the morning of Monday, June 4th, I met, by appointment, some twenty-five to thirty of the Santee Indians, who desired to express their thanks for the efforts in their behalf of friends in the East during the past winter, and also to bring to my attention a matter which they were desirous of calling to the notice of the Indian Rights Association. It seems from their statement, and from that of others with whom I conversed, that the Rev. Mr. Fowler, in the course of his ministerial work among the Indians, had been in the habit of giving homœopathic medicines to such persons as requested them, and in certain cases, when called, of visiting the sick and prescribing for them. This practice was *objected* to by the *resident physician* as an interference with his prerogative and an injury to his professional success! The matter was examined by Mr. Lightner, the agent, and through his representations an order was procured from the Department prohibiting Mr. Fowler from further distribution of medicine. [Free country, eh!] Since then Mr. Fowler has strictly adhered to the order of the Department and has refused, apparently much to the regret of the Indians, any further use of his remedies among them. The Indians who held conference with me considered the prohibition tyrannical, and maintained that in depriving them of Mr. Fowler's medicines, which had been of great benefit to their wives and children, their rights were invaded and they were compelled to suffer hardship. In my reply to them on this question, I briefly stated that it was a matter of regret to me if they had been forbidden the use of such medicines as had proved beneficial to them, and that I would refer the matter to the judgment of their friends in Philadelphia. With this they were apparently satisfied, and our meeting adjourned."

